# Intense interaction between biochar/g-C_3_N_4_ promotes the photocatalytic performance of heterojunction catalysts

**DOI:** 10.1039/d4ra03232b

**Published:** 2024-06-19

**Authors:** Rundong Ma, Yihui Sun, Hui Zhang, Jie Zhu, Han Tian, Xiong Guo, Ruifen Wang, Xiangzhi Cui, Xinmei Hou, Shengli An

**Affiliations:** a Shanghai institute of Ceramics, Chinese Academy of Sciences Shanghai China cuixz@mail.sic.ac.cn; b Beijing University of Science and Technology, Carbon Neutrality Institute Beijing China; c School of Materials and Metallurgy Inner Mongolia University of Science and Technology Baotou China

## Abstract

In recent decades, environmental protection and energy issues have gained significant attention, and the development of efficient, environmentally friendly catalysts has become especially crucial for the advancement of photocatalytic technology. This study employs the sintering method to produce biochar. A hybrid photocatalyst for the degradation of RHB under visible light was prepared by loading varying proportions of biochar onto g-C_3_N_4_ using ultrasonic technology. Among them, 2% CGCD (2% biochar/g-C_3_N_4_) achieved a degradation rate of 91.3% for RHB after 30 minutes of visible light exposure, which was more than 25% higher than GCD (g-C_3_N_4_), and exhibited a higher photocurrent intensity and lower impedance value. The enhancement in photocatalytic activity is primarily attributed to the increased utilization efficiency of visible light and the electron transfer channel effect from a minor amount of biochar, effectively reducing the recombination of photo-generated charge carriers on the g-C_3_N_4_ surface, thereby significantly improving photocatalytic activity. The degradation of RHB is synergistically mediated by O_2_^−^, h^+^ (photo-generated holes), and ˙OH. The free radical capture experiment indicates that O_2_^−^ and ˙OH are the primary active components, followed by h^+^.

## Abstract 2

Biochar/g-C_3_N_4_ composite photocatalyst was prepared using ultrasonic composite method. The conductive biochar acts as a charge transfer channel, effectively suppressing the recombination of photo generated carriers in g-C_3_N_4_.

## Graphical abstract

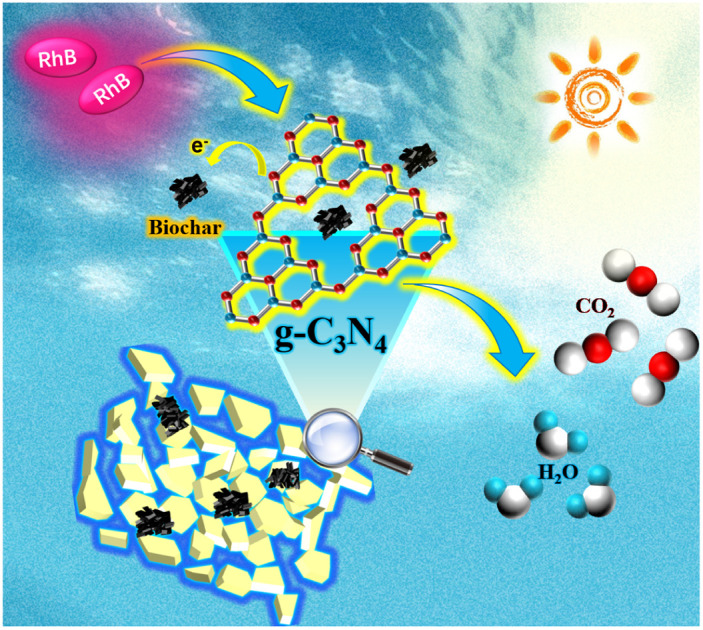

## Introduction

With the rapid development of the global economy and technology, the accompanying environmental pollution and energy crisis have become focal points of human concern. In the vast energy system of nature, solar energy is inexhaustible, which also propels the advancement of cutting-edge fields such as the semiconductor industry and photovoltaic technology. Among them, photocatalysis technology utilizes solar energy as its energy source, effectively addressing the energy crisis.^1–3^ Therefore, the key to photocatalysis technology lies in the development of low-cost, efficient, and stable photocatalysts.

Graphite phase carbon nitride (g-C_3_N_4_) is an excellent N-type semiconductor with stable physical and chemical properties, high surface charge density, which makes it easier to modify and regulate.^4^ Its two-dimensional layered structure similar to graphene gives it a large specific surface area, which also lays a good advantage in the field of photocatalysis.^5,6^ However, g-C_3_N_4_ suffers from poor visible light absorption ability, large bandgap width, and high recombination rate of photo generated charge carriers, greatly limiting its effective application in the field of photocatalysis.^7,8^ At present, many researchers have carried out a series of modification works on g-C_3_N_4_,^9–11^ such as microstructure control, heterojunction loading/construction, element doping, *etc*.^12,13^ Dai *et al.* prepared g-C_3_N_4_/Ag_3_VO_4_ composite materials containing Ag nanoparticles using chemical deposition method, and tested the photocatalytic degradation effect of MB under visible light. The results showed that the optimal photocatalytic performance was achieved when the ratio of Ag_3_VO_4_ to g-C_3_N_4_ was 5 : 2. The improvement of photocatalytic performance of composite materials is due to the strong coupling heterojunction formed between Ag_3_VO_4_ and g-C_3_N_4_. In addition, the porous structure of g-C_3_N_4_ and the surface plasmon resonance (SPR) effect of Ag accelerate the separation and transport of photo induced electron hole pairs, and effectively reduce carrier recombination.^14^ The composite modification of noble metal nanoparticles and ion doping can effectively reduce the recombination rate of g-C_3_N_4_ carrier and greatly improve photocatalytic efficiency. However, the preparation of this type of composite catalyst typically requires electrochemical deposition and hydrothermal methods, which may increase the cost of catalyst preparation and reduce the yield of the catalyst. Meanwhile, charge transfer at heterogeneous interfaces is often considered an important reason for the improvement of catalyst activity, and there is currently limited progress in this research.

Owing to the low loading/doping costs, as well as their ease of scaling up and industrialization.^15^ Carbon-based functional materials are playing a vital role in environmental protection, electrochemistry, and photocatalysis,^16^ with vast research opportunities ahead.

Biochar is the product of high-temperature pyrolysis of biomass under anaerobic conditions. Due to its low cost, environmental friendliness, and ease of preparation, biochar has also been widely used in various research fields, such as catalysis and the adsorption of heavy metal ions in soil.^17,18^ The graphite structure in biochar also endows it with good photoelectric properties. Luo *et al.* prepared tea residue biochar/g-C_3_N_4_ composite photocatalyst using high-temperature calcination method for reducing uranium in water.^19^ The results show that the removal rate of U(vi) by the composite photocatalyst TBC/g-C_3_N_4_ composite material can reach 99.64%, which is much higher than that of pure g-C_3_N_4_ (58.8%). The photogenerated electrons on the conduction band of g-C_3_N_4_ transitioning to TBC can effectively delay the recombination of g-C_3_N_4_ photogenerated carriers.^20^ Wang *et al.* prepared magnetism γ−. The catalytic performance of Fe_2_O_3_/O_2_-g-C_3_N_4_/BC composite material was tested. The results showed that the composite photocatalyst can rapidly and completely degrade the antibiotic sulfamethoxazole (SMX), with a mineralization rate of up to 62.3%. Sulfates and hydroxyl radicals are the main species in photocatalytic reactions.^21^

In this article, we prepared biochar using calcination method under anaerobic conditions, and prepared biochar/g-C_3_N_4_ (*x*% CGCD, *x* = 1, 2, 3, 5) composite catalyst with easy amplification and simple operation using ultrasonic composite method. Then, we characterized the microstructure of the prepared catalyst, tested its photoelectrochemical properties, and applied the catalyst to the degradation of RHB. The characteristics and advantages of composite catalysts were comprehensively analyzed from the perspectives of charge transfer, utilization of light and bandgap transformation, as well as the degradation mechanism of RhB.

## Results and discussion

### Powder biochar preparation

Put 300 g of wheat straw powder sieved with a 100-mesh sieve into a cylindrical crucible, fill the muffle furnace with heat-resistant bricks, and fix the crucible to maintain its airtightness. Gradually increase the temperature to 500 °C at a rate of 3 °C min^−1^ and hold for 2 hours, then remove the sample and label the resulting black powder (biochar) as BC. Soak and clean the black biochar powder BC obtained by washing with 2 M hydrochloric acid, and further wash repeatedly with ethanol and deionized water.

### Powder g-C_3_N_4_ preparation

Weigh 40 grams of urea (CH_4_N_2_O), place it in a 200 milliliter corundum crucible, seal it with tin foil, and place it in a muffle furnace. Gradually heat it up to 550 °C at a heating rate of 5 °C min^−1^, maintain it for 240 minutes, and cool naturally to room temperature. Take out the sample and grind it thoroughly to obtain a light-yellow powder of g-C_3_N_4_, which is labeled as GCD.

### Composite photocatalysts preparation

**Fig. 1:**
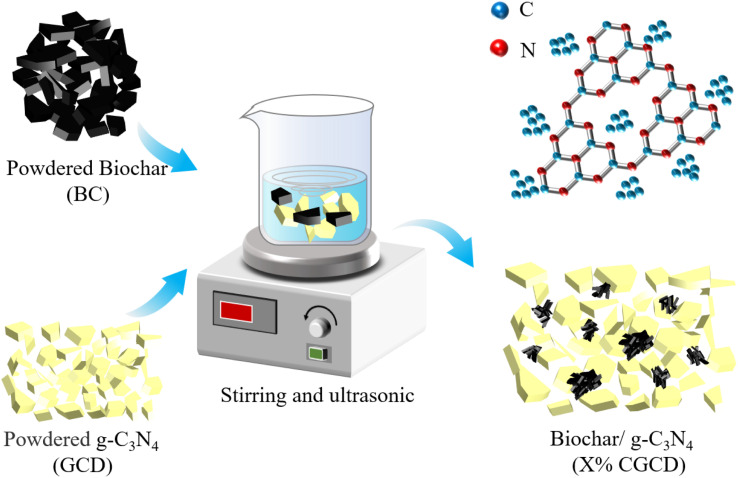
Flow chart for the preparation of CGCD.

As shown in Fig. 1, the biochar to g-C_3_N_4_ ratios is adjusted to 1, 2, 3, and 5 : 100, respectively. The particles were dispersed in 30 mL of ethanol and 40 mL of deionized water using ultrasound for 2 hours, then stirred with a magnetic stirrer for 2 hours to obtain a biochar/g-C_3_N_4_ composite photocatalyst, denoted as *X*% CGCD (*X* = 1, 2, 3, 5).

## Photocatalytic experiment design

### Activity testing

Adding 30 mg of photocatalyst to 50 mL of RHB solution with a concentration of 20 mg L^−1^, stirring the photocatalyst under no light conditions for 30 minutes to achieve adsorption desorption equilibrium, and absorb the supernatant to measure its absorbance *A*. Open the visible light disc (with a light intensity of 260 mW cm^−2^) for degradation experiments, and extract 4 mL of the upper clear liquid every 10 minutes before centrifugation for 10 minutes. Test its *A* value at 554 nm and take a total of 7 samples. The degradation efficiency of RHB is measured by the following formula.

1 *η* = (*A*_0_ − *A*)/*A*_0_ × 100% = (*C*_0_ − *C*)/*C*_0_ × 100%

In the eqn (1), *η* represents the photocatalytic degradation efficiency, *A*_0_ represents the initial absorbance of RHB, *A* represents the absorbance at the time of sample removal, *C*_0_ is the initial concentration, *C* is the concentration value of RHB at different time periods. Typically, *A* value is the absorbance of RHB at 554 nm, which is the maximum absorbance of RHB solution. This is also used to distinguish RHB from other organic compounds. The photocatalytic performance of the sample was tested using the Beijing Perfect light PCX-50C multi-channel photochemical reactor, with a light intensity of 260 mW cm^−2^.

### Cyclic performance testing and free radical capture experiment

The remaining suspension in the reaction flask after the light dark reaction is collected, filtered, washed, and then collected for the second photocatalytic reaction experiment. The reaction stability of the catalyst is evaluated by a total of five cycles of tests. 30 mg BQ (O_2_^−^), 30 mg KI (h^+^), and 20 mL TBA (˙ OH) free radical collectors are added to participate in the carrier capture experiment, and further explore the mechanism of the photocatalytic reaction.

## Crystalline phase and morphology of materials

**Fig. 2:**
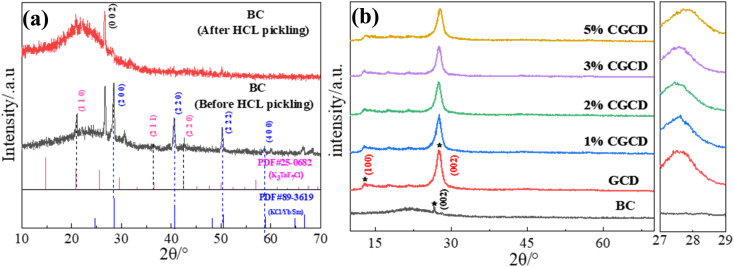
XRD spectra of different samples. (a) XRD comparison of wheat straw biochar before and after acid leaching; (b) XRD patterns of different samples.

The XRD diagram of the sample is shown in Fig. 2. Among them, Fig. 2a shows the XRD diagram of wheat straw biochar. Biochar without acid washing, ethanol, and deionized water treatment contains a large amount of ash and exhibits high diffraction peaks. From the perspective of composition, the ash is mainly composed of inorganic salts and some metal elements. After acid washing treatment, the diffraction peak of amorphous at around 22° of wheat straw biochar is more obvious, indicating a more orderly and compact arrangement of its C structure. The diffraction peak at around 27° is the (002) crystal plane of graphite, which is likely the ordered carbon crystal structure after cellulose combustion. Fig. 2b shows the XRD spectrum of the composite photocatalyst. The characteristic peaks around 13.1° and 27.3° represent the in-plane stacking and interlayer stacking of triazine rings in g-C_3_N_4_, respectively.^22,23^ From the graph, it can be observed that as the amount of biochar introduced increases, the spacing between the (002) crystal planes of g-C_3_N_4_ decreases, and the half peak width increases, meanwhile, the diffraction peak of the (002) crystal plane of g-C_3_N_4_ shifts towards higher angles. Which suggest there may be a strong interaction between biochar and g-C_3_N_4_, leading to a significant reduction in the interplanar spacing of g-C_3_N_4_ and grain refinement, which in turn diminishes agglomeration in g-C_3_N_4_.

**Fig. 3:**
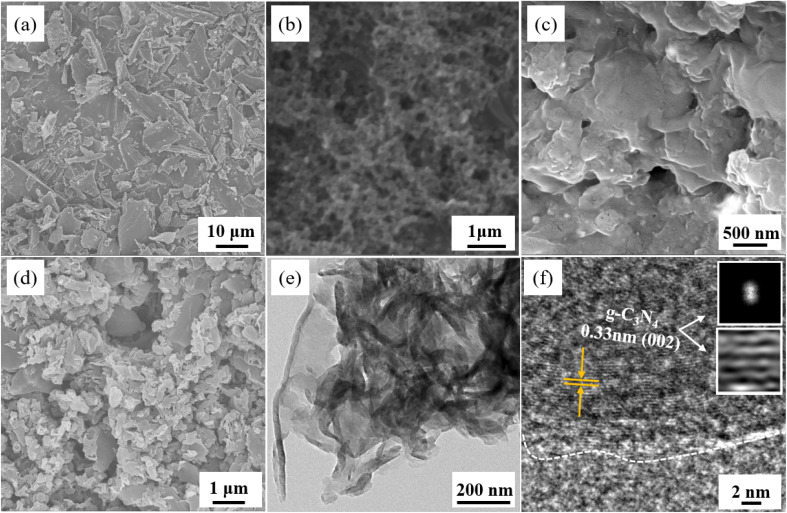
SEM (a–d) and HR-TEM (e and f) morphology of different samples; (a) BC (before HCL pickling); (b) BC (after HCL pickling); (c) GCD; (d) 2% CGCD; (e and f) HR-TEM morphology and lattice spacing of 2% CGCD.

Fig. 3 shows the SEM and HR-TEM images of different samples. Wherein, Fig. 3a shows the powdered biochar obtained by direct grinding after calcination and preparation, which presents an irregular block shape; Fig. 3b shows the biochar washed *via* HCL, and its surface exposes a rich microporous structure, which indicates that the corrosion of hydrochloric acid can effectively increase the specific surface area of biochar. Fig. 3c shows the microscopic morphology of g-C_3_N_4_ (GCD). The results indicate that the sample exhibits a block/layered structure formed *via* the stacking of nanosheets, which is formed *via* the gradual stacking and recombination of g-C_3_N_4_ molecules. Fig. 3d shows the microstructure of 2% CGCD, which can be seen to be formed *via* the continuous stacking of nanoparticles or nanosheets of varying sizes. Fig. 3e and f shows the HR-TEM images of 2% CGCD, and it can be observed that the sample after ultrasonic treatment still maintains a nanosheet morphology. In Fig. 3f, lattice stripes on the (002) crystal plane of g-C_3_N_4_ in the composite sample can be observed, with a crystal plane spacing of 0.33 nm. This also proves the good mechanical stability of g-C_3_N_4_, which is consistent with the analysis of XRD results.

**Fig. 4:**
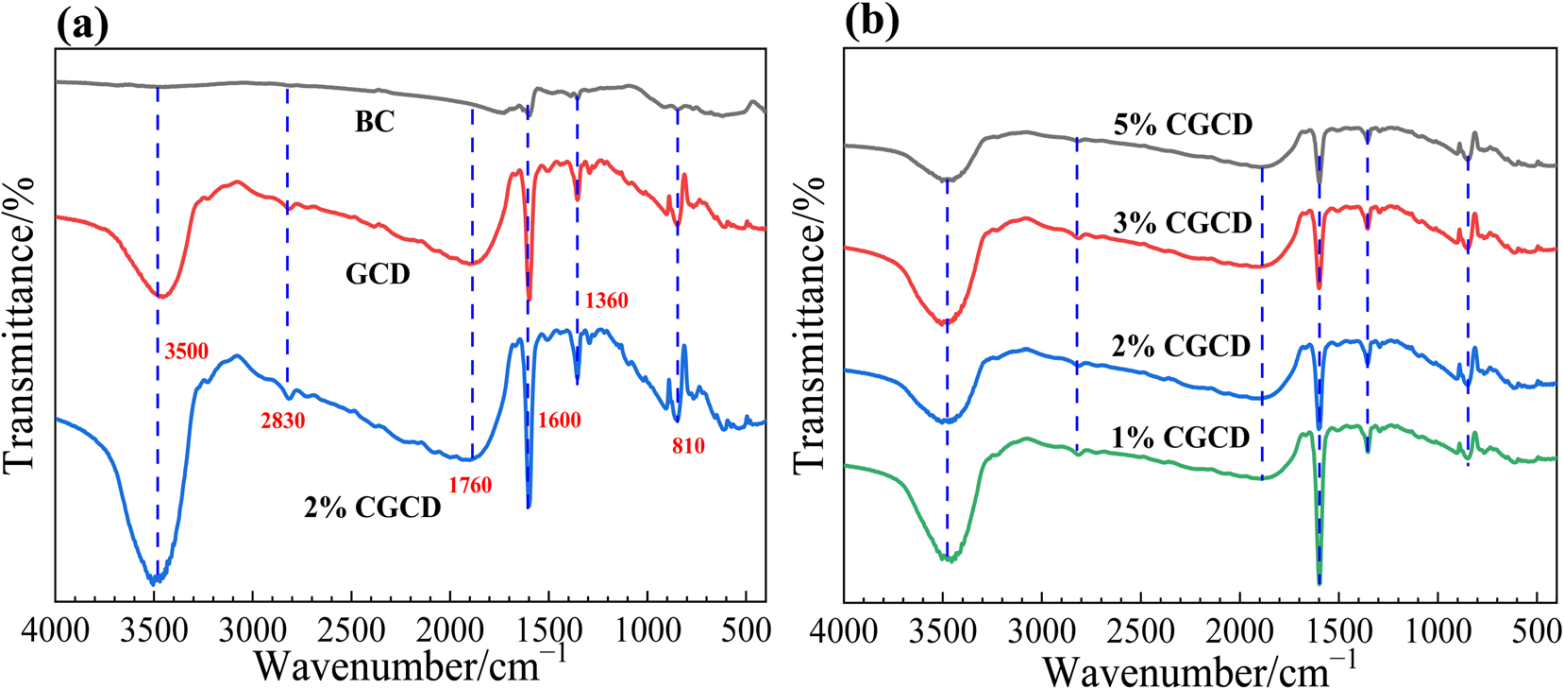
FT-IR spectra of different samples; (a) FT-IR diagrams of different samples before and after recombination; (b) FT-IR spectra of composite photocatalysts.

The functional groups of the sample were characterized using FT-IR spectroscopy. As depicted in Fig. 4a, the reflection peak at 2820 cm^−1^ is attributed to the stretching vibration of the relevant N–H bonds, which belong to the non-condensed NH_2_ groups.^24,25^ The reflection peak at 3500 cm^−1^ is due to the vibration (surface adsorption) of hydroxyl (−OH) in the associated state,^26^ likely resulting from the adsorbed oxygen formed by the sample's interaction with ethanol and water molecules during ultrasonic treatment. This peak range is absent in the biochar's spectral profile, likely attributed to the binding of H^+^ with surface hydroxyl groups during the acid leaching process, or it may be related to the size of biochar pores. As shown in Fig. 4b, it is observed that as the biochar dosage increases, the intensity of the absorption peak at 1600 cm^−1^ of the heptane triazole ring varies, signifying that the introduction of biochar alters the chemical environment surrounding g-C_3_N_4_, indicating successful composite formation between the two samples.

**Fig. 5:**
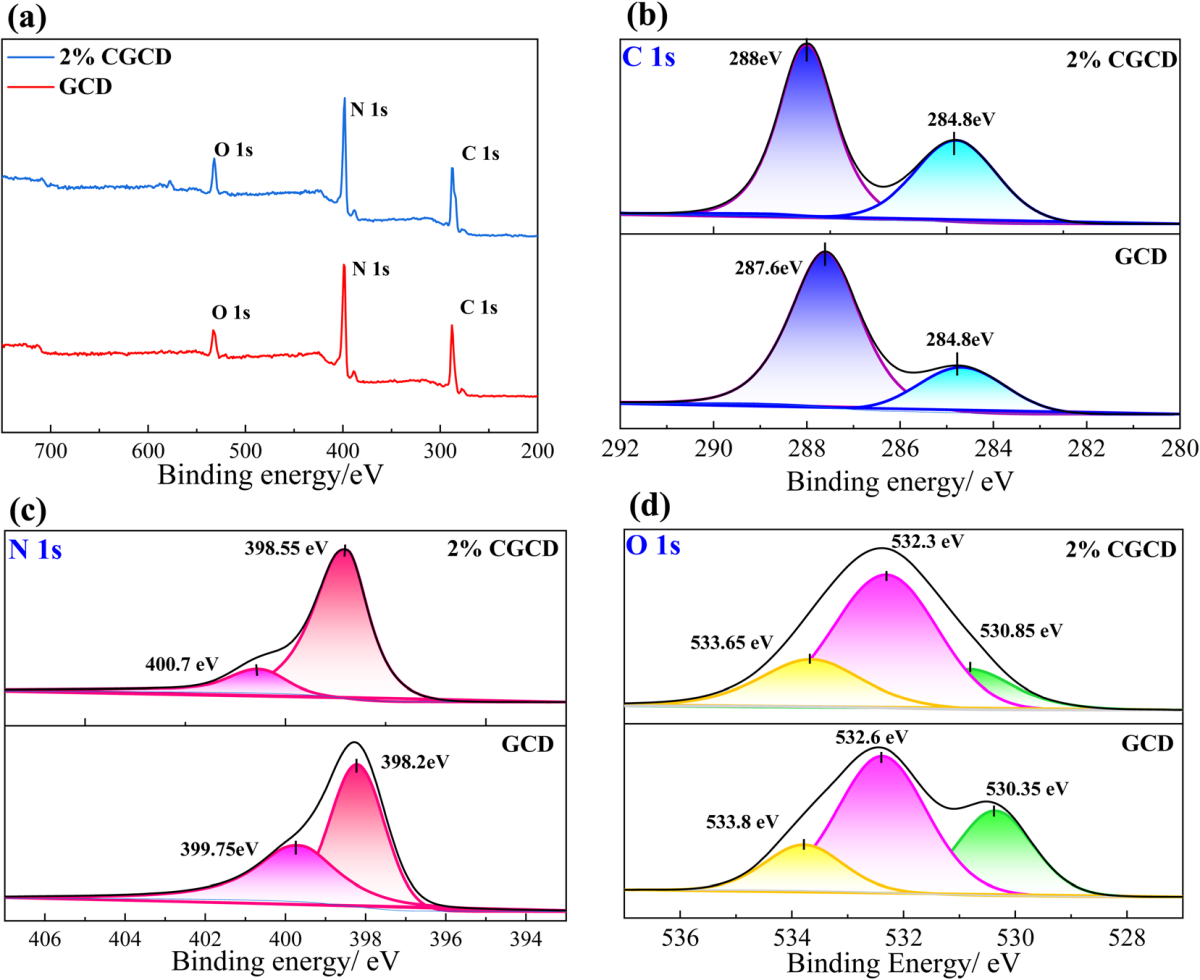
XPS spectrum of different samples. (a) Full spectrum; (b) C_1s_; (c) N_1s_; (d) O_1s_.

The surface elemental composition of GCD and 2% CGCD was analysed by using XPS. Fig. 5a displays the total spectrum of the photocatalyst, revealing three elements, C, N, and O, in the XPS spectrum. Notably, 2% CGCD has a larger O peak area, indicating an increase in oxygen atoms adsorbed on the surface of the composite sample. Fig. 5b shows the spectral peak of the C 1s energy level in the sample. Among them, the binding energy at 287.6 eV corresponds to the N–C=N characteristic peak of sp^2^ hybrid C in GCD, the peak at 398.2 eV in Fig. 5c corresponds to the C=N–C structure in GCD,^27^ and the peak at 399.75 eV corresponds to the –NH_2_ group of non-polymerizations, which together form triazine ring structural units. From Fig. 5b and c, it can be seen that in the composite sample, all three peaks shift towards higher chemical shifts, indicating the presence of charge transfer on the surface of the triazine ring, which mainly shows a trend of losing electrons. This indicates that biochar, as a charge transfer channel, can attract electrons from the conduction band of GCD, and the two exhibit strong interactions. As shown in Fig. 5d, the positions at 530.35 eV, 532.6 eV, and 533.8 eV correspond to the C=O, C–O, and O–C (O) bonds, respectively. Among them, the C=O content in 2% CGCD is significantly lower than that in GCD, and the binding energy undergoes a red shift, indicating that ultrasound method has a destructive effect on the C=O bond, which is more conducive to the recombination of BC and GCD.^28^

**Fig. 6:**
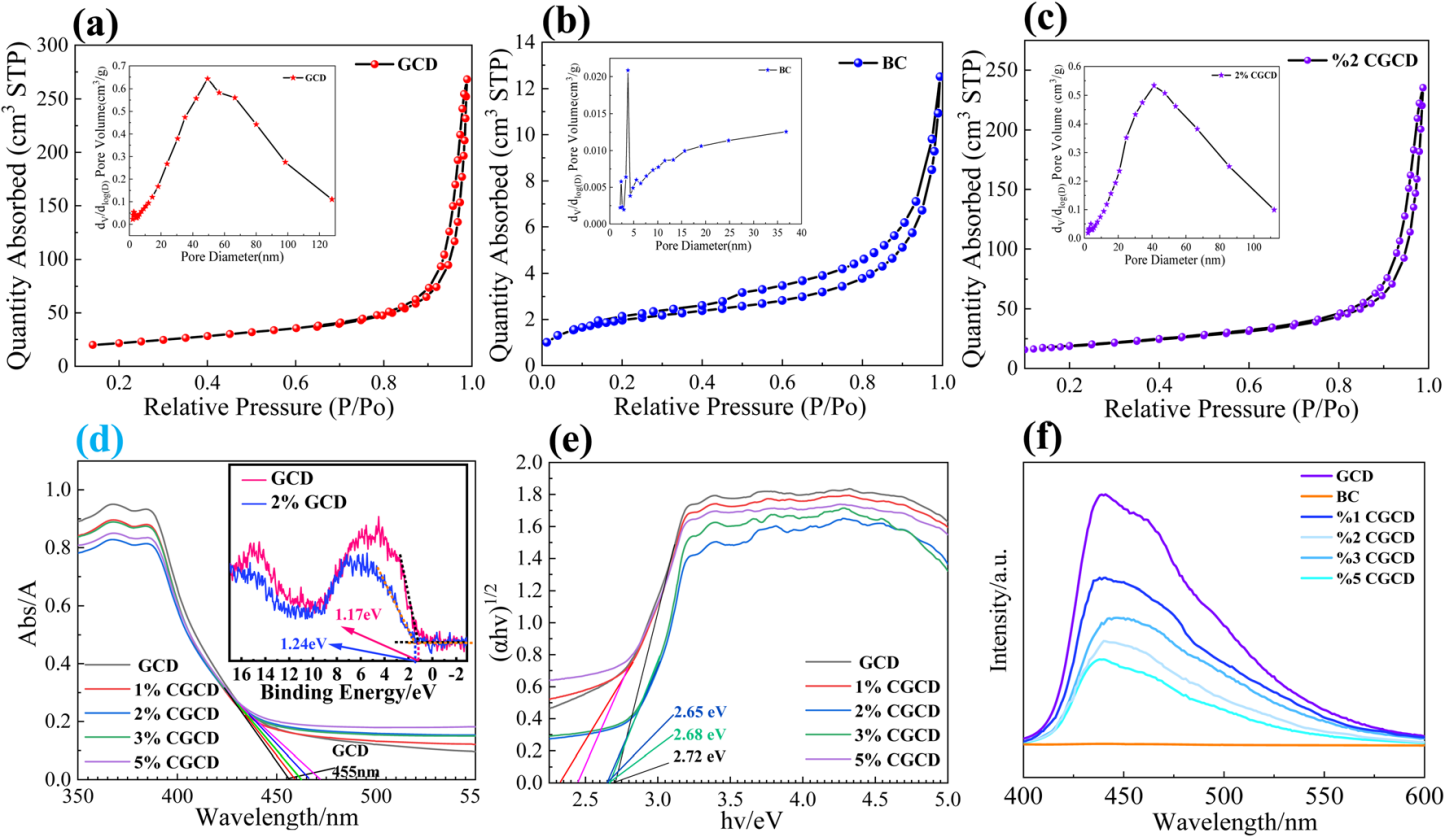
N_2_ isothermal adsorption–desorption curves and pore size distribution of different samples: GCD (a); BC (b); 2% CGCD (c). UV-vis spectra and valence band spectrum of different samples (d); Tauc-curve (e); PL spectra (f).

N_2_ adsorption desorption and pore size distribution tests were conducted on different samples. As shown in Fig. 6a–c, the pore types of powdered GCD are mostly mesoporous structures, with a pore size of approximately 48 nm; The pore size of 2% CGCD is approximately 40–50 nm, slightly larger than that of GCD. This can be attributed to the fragmentation and formation of smaller particles and nanosheets in bulk GCD under ultrasound action. BC has abundant micropores and a small amount of mesoporous structure. Among them, the pore size of the micropores is about 3–4 nm, which may be attributed to the pores left by the evaporation of water molecules during the combustion of lignin and cellulose in wheat. The mesoporous pore size is approximately 30–40 nm, which may be attributed to the collapse and merging of micropores. All three samples have hysteresis loops, and the shape of the pores is mostly H4-type fine pores formed by the stacking of layered molecules, which is consistent with the characterization results of SEM and XRD. Table 1 shows the statistical distribution of sample specific surface area. It can be observed that the specific surface area of GCD located between biochar and GCD is about 73.9 m^2^ g^−1^, and the specific surface area of the composite photocatalyst sample is about 66.7 m^2^ g^−1^. This may be related to the shortening of the spacing between GCD crystal planes in the composite sample, indicating the successful recombination of the two samples.^29^

**Table 1:** Specific surface area of different samples

Sample name	GCD	BC	2% CGCD
BET surface area (m^2^ g^−1^)	73.87	7.10	66.73

As shown in Fig. 6d, the absorption edge of pure graphite phase carbon nitride is about 455 nm. As the amount of biochar introduced increases, the absorption band edge gradually shifts to red, indicating a significant improvement in the response of the photocatalyst to visible light. As shown in Fig. 6e, according to the formula *E*_g_ = 1240/*λ*, the estimated bandgap of g-C_3_N_4_ is approximately 2.72 eV. Meanwhile, according to the Tauc plot formula, a Tauc plot is drawn, which also satisfies this condition, the Tauc plot also satisfies this condition The position of the valence band (VB) and conduction band (CB) of GCD can be calculated according to formulas (2) and (3). In the formula, Ec is the dipole moment of the standard hydrogen electrode, which is 4.5 eV in value, E_g_ is the band gap width of the semiconductor, and E_CB_ and E_VB_ represent the conduction band and valence band values of the semiconductor, respectively. *X* is the absolute electronegativity of the semiconductor, which is numerically equal to the geometric average value of each component atom in the semiconductor. Through consulting the literature, the empirical value of XGCD is 4.73,^30^ and further calculation shows that its *E*_VB_ and *E*_CB_ are 1.13 eV and −1.59 eV, respectively. In order to further refine the band data, we conducted XPS valence band spectrum testing, the top energy of GCD's CB is 1.17 eV, which is close to the calculated value (Fig. 6d). With the increase in biochar, the band gap of the composite photocatalyst is shorter than that of pure g-C_3_N_4_, and the absorption band edge is gradually red shifted, indicating that the introduction of biochar enhances the response of the g-C_3_N_4_ system to visible light, and shortens its band gap, which is more conducive to the transition of photogenerated carriers and increases the charge density of the catalyst surface, thus improving the activity of the photocatalyst.

2 *E*_CB_ = *X* − *E*_C_ − *E*_g_/2

3 *E*_VB_ = *E*_CB_ + *E*_g_

The recombination of photo-generated carriers in the sample was analysed *via* PL fluorescence spectroscopy. As shown in Fig. 6f, when visible light's energy is greater than the sample bandgap used to irradiate the sample, the photo-generated electrons undergo energy level transitions. When photo-generated charge carriers recombine, some of the energy is released in the form of phonons, manifested as lattice vibrations, while the other part is released in the form of photons (fluorescence).^31^ From the figure, it can be seen that with an excitation of 370 nm wavelength light, the carrier recombination rate of 2% CGCD is significantly lower than that of pure graphite phase carbon nitride. Biochar does not produce fluorescence in this band, indicating that the introduction of biochar significantly reduces the recombination of g-C_3_N_4_ carriers, which helps to efficiently carry out photocatalytic reactions.

### Photocatalytic performance test

**Fig. 7:**
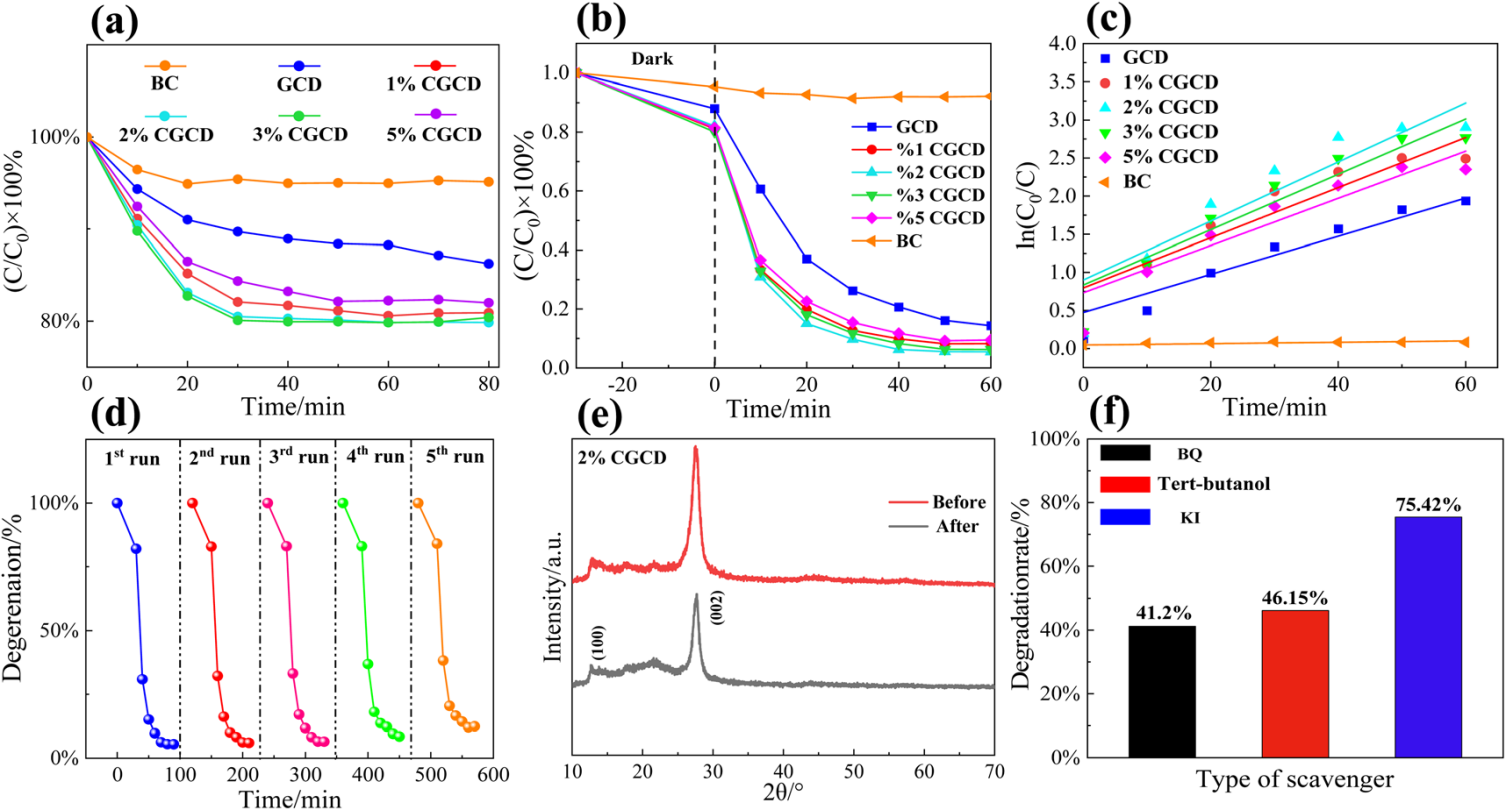
(a) Adsorption experimental plots of different samples; (b) degradation rate of different photocatalyst samples; (c) fitting diagram of degradation kinetics for different samples; (d) cyclic degradation rate of 2% CGCD with time; (e) XRD patterns of 2% CGCD before and after circulation experiment; (f) pattern of free radical trapping experiment.

Evaluate the reaction activity of photocatalysts based on the degradation rate of RHB. Firstly, we tested the adsorption activity of different samples on RHB, and the catalyst dosage and dye concentration in this adsorption experiment were the same as those in the photocatalytic activity experiment. As shown in Fig. 7a, with the passage of reaction time, RHB is gradually adsorbed by the catalyst. When the reaction time is 30 minutes, all samples are close to adsorption equilibrium, and the degradation rate of RHB no longer changes significantly with time. Based on the results of adsorption experiments, the photocatalytic activity of different samples was tested. As shown in Fig. 7b, the degradation efficiency of RHB by different samples is 2% CGCD > 3% CGCCD > 1% CGCCD > 5% CGCCD > GCD. Among them, 2% CGCD reacted under light for 30 minutes, and the degradation rate of RHB was 91.3%, which was 25% higher than GCD. The composite sample exhibits stronger photocatalytic activity than GCD, which may be due to the introduction of biochar enhancing the catalyst's absorption of visible light and improving the utilization efficiency of visible light. This may also be due to the shortened bandgap of the composite sample, the reduced resistance of the photo to the generated electronic transitions, and the increased surface charge density of the composite sample. It is worth noting that g-C_3_N_4_ composite with biochar exhibited excellent degradation effects on RhB during the dark reaction stage, which means electrons in g-C_3_N_4_ transfer to biochar and are in a charge deficient state, which also helps to enhance the oxidation of g-C_3_N_4_ and enhance the oxidation reaction with the adsorbed RhB, thereby accelerating the adsorption degradation kinetics of RhB.^32^ Meanwhile, crystalline graphite in biochar can also serve as a charge transfer channel, promoting the spatial separation of photo generated charges at the conduction band of GCD, thereby reducing the recombination rate of photo generated carriers in GCD and placing GCD in a charge deficient state, enhancing its oxidizing ability. The degradation reaction data was fitted using quasi first-order kinetic linear regression, and the results are shown in Table 2 and Fig. 7c. The fitting curve is obtained from formula (4), where *C*_0_ is the initial concentration of RHB and C is the concentration of RHB at a certain moment in the hydrolysis reaction.^33,34^ The slope *k*_*t*_ represents the reaction rate constant of the first-order reaction. All data fitting regression coefficients *R*^2^ are above 0.87, and the order of reaction rates is 2% CGCD > 3% CGCCD > 1% CGCCD > 5% CGCCD > BC. Overall, with the increase of biochar content, the photocatalytic efficiency of the sample first increases and then decreases. When the content of biochar is 5%, the photocatalytic efficiency of the sample decreases, which may be due to the lack of photocatalytic activity of biochar itself. The increase in its content may cover the active sites of g-C_3_N_4_ and compete with it for photo generated electrons, resulting in a light shielding effect.^35^

4 ln(*C*_0_/*C*) = *k*_*t*_ + constant

**Table 2:** Reaction rate constant *k*_*t*_ and correlation coefficient *R*^2^ of samples

Sample name	GCD	BC	1% CGCD	2% CGCD	3% CGCD	5% CGCD
Rate constant (*k*/min^−1)^	2.53 × 10^−2^	0.9 × 10^−2^	3.29 × 10^−2^	3.88 × 10^−2^	3.63 × 10^−2^	3.09 × 10^−2^
*R* ^2^	0.929	0.877	0.914	0.908	0.925	0.924

Fig. 7d shows the cyclic performance test of 2% CGCD. After five cycles of experiments, the degradation rate of RHB in the sample can still reach 91.7%, indicating that the sample has good photocatalytic stability. The 2% CGCD sample after cycling was characterized *via* secondary XRD, as shown in Fig. 7e. After 5-times' cycling, the (100) and (002) crystal plane diffraction peaks of g-C_3_N_4_ in the sample did not show a red or blue shift, indicating that the sample can still maintain its stable phase structure after cycling testing. After cyclic testing, the diffraction peaks of the composite photocatalyst on the (100) and (002) crystal planes slightly decreased, which may be due to the slight damage and de-formation of the crystal state of the catalyst sample caused *via* ultrasound and washing. With *tert*-butyl alcohol, KI, and BQ as quenching agents, the photocatalytic degradation experiments of ˙ OH, h^+^, and O_2_^−^ were carried out, respectively. As shown in Fig. 7f, after the addition of *tert*-butyl alcohol, potassium iodide, and *p*-benzoquinone, the degradation rates of RHB *via* the composite photocatalyst de-creased to 46.15%, 75.42%, and 41.2%, respectively. It can be concluded that the main active species in the photocatalytic degradation experiment are ˙ OH and O_2_^−^, followed by h^+^. Due to the negative conduction band potential of GCD, it is more conducive to the reduction reaction of surface-adsorbed O_2_ and the generation of active species such as O_2_^−^ and ˙ OH. At the same time, GCD belongs to N-type semiconductors, and its surface has a high e^−^ density, while the density of h^+^ is relatively small, which may also make its oxidation effect less obvious.^36,37^

**Fig. 8:**
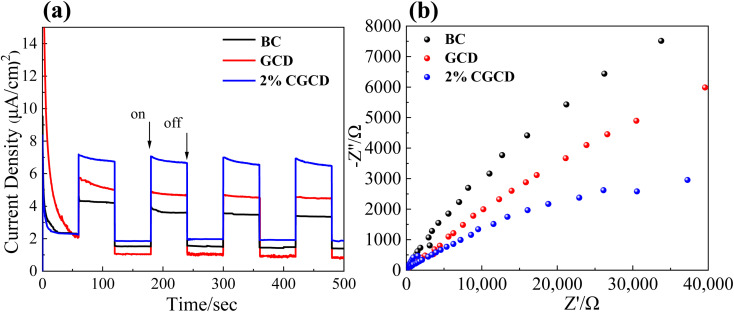
Electrochemical characterization of different samples; (a) electrochemical characterization test of composite photocatalysts; (b) electrochemical AC impedance spectroscopy.

To delve into the separation and recombination of charge carriers in biochar/g-C_3_N_4_ composite samples, transient chronoamperometry and electrochemical impedance spectroscopy were applied to the catalyst before and after the recombination process. As depicted in Fig. 8a, the transient current intensity of 2% CGCD is notably greater than that of GCD, implying that the composite photocatalyst boasts an outstanding level of photo-induced carrier separation, thereby facilitating carrier migration at the membrane electrode/electrolyte interface. The arc radius on the EIS spectrum is linked to the charge transfer at the interface between the working electrode and electrolyte,^38^ as illustrated in Fig. 8b. The arc radius of the EIS electrode reflects the charge transfer resistance in the electrode/electrolyte solution, with a larger radius corresponding to a higher charge transfer resistance. Notably, the charge transfer resistance of 2% CGCD is significantly reduced compared to that of pure graphite phase carbon nitride, indicating that the composite sample exhibits reduced electrode/electrolyte transfer resistance and enhanced charge transfer capability.

### Mechanism investigation and discussion

**Fig. 9:**
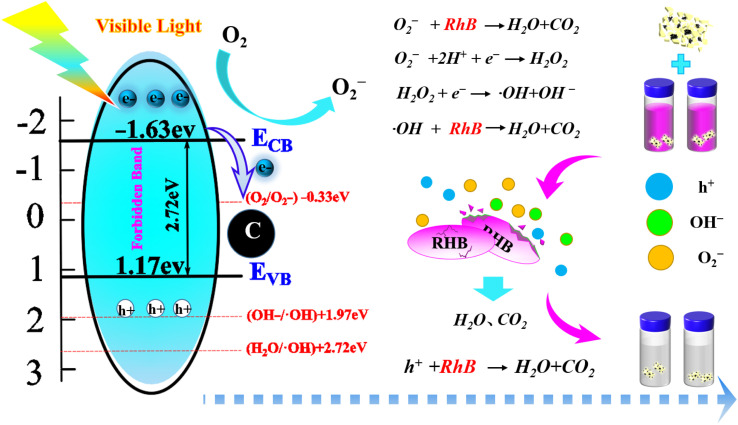
Schematic diagram of the mechanism of photocatalytic degradation of RHB.

To further elucidate the reaction mechanism behind the degradation of RHB by reactants, we computed the valence band potential and conduction band potential of GCD using the energy band formula, which were found to be 1.17 eV and −1.59 eV, respectively. Photocatalytic mechanism was followed by a comprehensive analysis in conjunction with radical trapping experiments. As depicted in Fig. 9, the degradation of RHB is primarily categorized into three pathways. The first type involves redox reactions between RHB and the exposed photo-generated holes on the catalyst surface. Upon exposure to visible light, photo-generated electrons transition from the valence band of GCD to the conduction band, generating oxidative photo-generated holes. These holes, by their own oxidation properties, oxidize RHB and breaking its benzene ring and disrupting its C=C structure, ultimately mineralizing RHB into H_2_O and CO_2_.^39^ The second type is the reduction reaction between the reducing electrons transitioning to the conduction band in photocatalysts and the catalyst surface in solution, yielding O_2_^−^ with strong redox properties. Owing to the unpaired electrons in O_2_^−^, it also exhibits strong redox characteristics and can mineralize and degrade RHB. In the third pathway, because of the N-type semiconductor properties of GCD, photo-generated electrons originate not only from the 2p orbital of C in GCD but also from the 2p orbital of N. As a result, the reduction reaction on the GCD surface is quite active, likely generating an excessive amount of O_2_^−^. O_2_^−^ can also react with H^+^ in water, producing a strong oxidizing and non-selective hydroxyl radical (˙OH), thereby oxidizing and degrading RHB. During the degradation process of RHB, photogenerated holes, O_2_^−^, and ˙OH exhibit a synergistic effect.

## Conclusions

In this article, a simple method was used to calcined corn straw into conductive biochar, and the ultrasound stirring co mixing method, which is easy to amplify, was used to prepare biochar/g-C_3_N_4_ heterojunction catalyst. Composite catalysts exhibit excellent adsorption and photocatalytic activity for RhB degradation. The strong interaction between biochar and g-C_3_N_4_ promotes the electron transfer from g-C_3_N_4_ to biochar, leaving g-C_3_N_4_ in a charge deficient state, which promotes A's strong oxidizing ability and enables more efficient degradation of RhB. Among them, biochar serves as a co-catalyst.

## Author contributions

The author's contributions to this article are as follows: writing – original draft: Rundong Ma. Writing – review & editing: Rundong Ma, Xiangzhi Cui, Han Tian. Funding acquisition, resources: Xiangzhi Cui. Supervision: Xiangzhi Cui, Han Tian, Ruifen Wang, Xinmei Hou, Shengli An. Formal analysis: Yihui Sun, Hui Zhang, Jie Zhu, Xiong Guo. Conceptualization: Rundong Ma, Xiangzhi Cui. We thank all authors for their contributions to this article.

## Conflicts of interest

There are no conflicts to declare.
